# A convenient separation strategy for fungal anthraquinones by centrifugal partition chromatography

**DOI:** 10.1002/jssc.202100869

**Published:** 2022-01-19

**Authors:** Fabian Hammerle, Michael Zwerger, Anja Höck, Markus Ganzera, Ursula Peintner, Bianka Siewert

**Affiliations:** ^1^ Institute of Pharmacy, Pharmacognosy Center for Molecular Biosciences Innsbruck University of Innsbruck Innrain 80/82, Innsbruck Tyrol 6020 Austria; ^2^ Institute of Microbiology University of Innsbruck Technikerstraße 25, Innsbruck Tyrol 6020 Austria

**Keywords:** anthraquinone glycosides, anthraquinones, basidiomycetes, centrifugal partition chromatography, cortinarius fruiting bodies

## Abstract

As recently shown, some fungal pigments exhibit significant photoactivity turning them into promising agents for the photodynamic treatment of microbial infections or malignant diseases. In the present study, a separation strategy for fungal anthraquinones was developed based on centrifugal partition chromatography. A suitable method was explored employing a methanolic extract of the fruiting bodies of *Cortinarius sanguineus* (Agaricales, Basidiomycota). An excellent fractionation was achieved using a biphasic solvent system comprising chloroform/ethyl acetate/methanol/water/acetic acid (3:1:3:2:1, v/v/v/v/v) operating in ascending mode. Experiments on an analytical scale with extracts of closely related *Cortinarius* species exhibited broad applicability of the devised system. Up to six pigments could be purified directly from the crude extract. Preparative‐scale fractionation of the methanol extracts of *C. malicorius* and *C. sanguineus* demonstrated that up‐scaling was possible without compromising selectivity.

**Article Related Abbreviations**
AQ: anthraquinone
CPC: centrifugal partition chromatography
DMSO: dimethyl sulfoxide
LP: lower phase
MeOH: methanol
MLLP: multiple liquid–liquid partition
PT: pigmentation type
SI: electronic supplementary information
UP: upper phase

## 1 INTRODUCTION

Agaricoid fungi of the genus *Cortinarius* subgenus *Dermocybe* often have conspicuously colored fruiting bodies with auburn, orange, or even greenish‐yellow hues, which owe their striking appearance to the presence of anthraquinone (AQ) pigments [1]. After being used as fabric dyes in the 20th century [2], a previously overlooked bioactivity—i.e., photoactivity [3]—has brought these metabolites back into the spotlight. A dimeric AQ isolated from the marsh webcap (*Cortinarius uliginosus*) was shown to induce blue light‐dependent cell death in various cancer cell lines in the nanomolar range while being inactive in the absence of light [4]. Subsequently, several other fungal AQs (e.g., monomeric AQs, AQ carboxylic acids, dimeric AQs, and even glycosylated AQs) have been identified as promising photosensitizers [5].

One‐ and two‐dimensional thin‐layer chromatography (TLC) techniques are commonly used to isolate AQ aglycones on an analytical scale [6, 7]. However, preparative‐scale separation of these compounds by conventional column chromatography is severely impeded by their unfavorable physicochemical properties. Their low solubility, strong adsorption on silica gel, extensive tailing, and the resulting overlap of elution bands necessitate several labor intensive separation steps, each of which involves considerable loss of sample. Hynninen and Räisänen presented an elegant way to circumvent these problems by facilitating the isolation of natural AQs from *Cortinarius sanguineus* through multiple liquid–liquid partition (MLLP) with stepwise pH‐gradient elution [8]. Yet, their study was designed to isolate only aglycones by MLLP, as an enzymatic method with endogenous fungal β‐glucosidase was used to prepare their starting fractions [9]. In parallel, acidic hydrolysis followed by sequential crystallization steps was frequently utilized as a purification strategy for fungal AQs [10]. Recently, Wang and co‐workers applied high‐speed countercurrent chromatography to separate the perylenequinonoids pigments of *Shiraia bambusicola* [11]. In their native form, however, many fungal AQs are glycosylated [12]. Consequently, most fungal AQ glycosides of *Dermocybes* were only annotated and not isolated [1, 13]. Thus, an additional liquid–liquid separation technique is required to enable their separation and thorough chemical and biological characterization.

As a type of liquid–liquid chromatography, centrifugal partition chromatography (CPC) uses a biphasic immiscible liquid system without solid support for separation. Centrifugal forces retain the liquid stationary phase in the device while the mobile phase is pumped through. According to their partition coefficients, solutes are distributed and transferred between both phases [14]. Due to a variety of advantages, such as the complete recovery of injected components, the avoidance of irreversible adsorption and contamination of samples, as well as improved separation performance during scale‐up [15], CPC has established itself as a method of choice in natural product chemistry and has already been used for the separation of various compound classes across a broad polarity range [14, 16].

To develop a practical CPC method for the separation of AQs occurring in extracts of fungal fruiting bodies, e.g., from *Cortinarius* species, the present study involved (i) the development of a solvent system using a methanolic extract of *C. sanguineus* (i.e., a frequent species with high AQ content), (ii) its applicability to different fungal extracts, and (iii) scale‐up experiments.

## 2 MATERIALS AND METHODS

### 2.1 Chemicals

All solvents for the extraction procedures, HPLC analyses, and CPC separations were of analytical grade and sourced from VWR International (Vienna, Austria) and Merck (Merck, Darmstadt, Germany). Ethyl acetate was distilled prior to use. Ultrapure water was obtained using a Sartorius arium^®^ 611 UV purification system (Sartorius, Göttingen, Germany). TLC plates (ALUGRAM^®^ Xtra SIL G/UV _254_ 20 × 20 cm) were obtained from Macherey‐Nagel (Macherey‐Nagel, Düren, Germany).

### 2.2 General instrumentation

Weighing of samples was done with the balances Sartorius Cubis^®^‐series (Sartorius, Göttingen, Germany) and Mettler Toledo AB54 (Mettler‐Toledo, Gießen, Germany). The biomaterial was dried using a SP VirTris BenchTop Pro freeze dryer (SP Industries, Warminster, UK). The laboratory mill IKA Labortechnik MF10 basic (IKA^®^‐Werke, Staufen, Germany) equipped with a 0.5 mm sieve was used for the comminution of fungal biomaterial. The ultrasonic baths Sonorex RK 52 (BANDELIN electronic, Berlin, Germany) and Sonorex RK 106 as well as the heating bath GFL 1042 (Gesellschaft für Labortechnik, Burgwedel, Germany) were employed for the preparation of crude extracts. Evaporation of solvents was done using a Heidolph Laborota 4000 efficient rotary evaporator (Heidolph Instruments, Schwabach, Germany) coupled to a vacuubrand PC 101 NT (VACUUBRAND, Wertheim, Germany) vacuum pump. The Vortex‐Genie 2 vortex mixer (Scientific Industries, Bohemia, New York) was used to homogenize the samples. Pipettes and pipette tips were purchased from Eppendorf (Hamburg, Germany) or STARLAB International (Hamburg, Germany).

### 2.3 CPC apparatus and HPLC instruments

A laboratory‐scale CPC instrument from Kromaton Rousselet‐Robatel (KROMATON Sarl, Annonay, France) with a rotor volume of 55 mL was used for the preliminary investigations. The rotor consisted of 15 discs with 60 twin cells each, i.e., 900 twin cells in total. The size of the sample loop was 5 mL and the rotation speed was adjustable up to 3000 rpm. A Hitachi L‐7100 pump from Merck (Merck, Darmstadt, Germany) was used for solvent delivery. Eluting fractions were collected time‐dependently using the SuperFrac collector from Amersham Pharmacia Biotech (Uppsala, Sweden). For separations on a preparative scale, a Gilson CPC 1000 instrument coupled to a PLC 2250 unit (Gilson, Middleton, USA), which consisted of a quaternary pump and a DAD detector with a range of 200–600 nm, was employed. The total rotor capacity was 1000 mL, the rotation speed was adjustable up to 1500 rpm, and the sample loop volume amounted to 50 mL.

HPLC–MS experiments were performed on the modular system Agilent Technologies 1260 Infinity II equipped with a quaternary pump, vial sampler, column thermostat, diode‐array detector, and mass spectrometer (Agilent Technologies, Santa Clara, USA). The parameters for the MS data acquisition were set as follows: ionization source = API‐ES, drying gas flow = 12.0 L/min, nebulizer pressure = 1294 Torr, drying gas temperature = 320°C, and capillary voltage (negative ionization mode) = 4.5 kV. HPLC–DAD analyses were carried out on a Shimadzu LC‐20AD XR system (Shimadzu Europa, Duisburg, Germany) equipped with a DAD detector, autosampler, and column thermostat. A Synergi MAX‐RP 80 Å column (150 × 4.60 mm, 4 microns) from Phenomenex (Aschaffenburg, Germany) was used as the stationary phase. The mobile phase for all liquid‐chromatographic experiments comprised water (A) and acetonitrile with 0.1% formic acid (B). Elution was performed in gradient mode (0 min: 10% B, 3 min: 50% B, 5 min: 90% B, 7 min: 99% B, 11 min: 99% B, 11.1 min: 10% B, followed by 4 min of re‐equilibration with 10% B). The DAD was set to a detection wavelength of λ = 400 nm (additional wavelengths for purity calculations, SI, Table S4). The flow rate, sample volume, and column temperature were adjusted to 1.0 mL/min, 10 μL, and 40°C, respectively.

### 2.4 Fungal material, extract preparation, and metabolite annotation

The fruiting bodies of five *Cortinarius* species (i.e., *C. cinnabarinus*, *C. malicorius*, *C. olivaceofuscus*, *C. rubrophyllus*, and *C. sanguineus* var. *aurantiovaginatis*) were collected in various European countries between 1975 and 2020. Based on the usual macroscopic and microscopic techniques as well as rDNA ITS sequence analysis, a reliable taxonomic classification of the studied species was achieved (Table S1). We used *C. sanguineus* var. *aurantiovaginatus* in our study because it is the dominant variant in Austrian boreal *Picea abies* forests, while *C. sanguineus* var. *sanguineus* is very rare. For practical purposes, we address this as *C. sanguineus* in the text. Voucher materials from 2018 and older were dried with a dehydrator at 35–45°C. For more recent voucher materials (2019–2021), the fresh fruiting bodies were immediately frozen after collection and stored in a freezer (T = −18°C). Prior to extraction, these fruiting bodies were freeze‐dried, ground to a fine powder, and stored in small paper bags protected from moisture and direct light exposure. Voucher specimens of all species used in this study are deposited in the mycological collection of the Tiroler Landesmuseen (IBF | Naturwissenschaftliche Sammlungen | Tiroler Landesmuseen 2021 (tiroler‐landesmuseen.at)).

The dried fruiting bodies of *C. malicorius* (batch 2020) and *C. rubrophyllus* were extracted as follows: Finely ground biomaterial was extracted with methanol (V = 30 mL, n = 4) using an ultrasonic bath (5 min per extraction step) followed by filtration through a folded paper filter (particle retention: 10–20 μm). Respective filtrates were combined, solvents were removed by vacuum rotary evaporation at 40°C, and extracts were kept in a desiccator. The extraction of *C. malicorius* (m = 6014.2 mg) and *C. rubrophyllus* (928.5 mg) yielded 1269.8 mg (21.1% w/w), and 351.8 mg (37.9% w/w) of crude extract, respectively. The dried and milled carpophores of *C. sanguineus* (m = 34.8 g) were extracted successively with petroleum ether (V = 500 mL, n = 4), dichloromethane (V = 500 mL, n = 6), and methanol (V = 500 mL, n = 10) utilizing ultrasonication (5 min per extraction step). After each extraction step, filtration was performed, and the respective filtrates were combined and dried using rotary evaporation in vacuo at 40°C. The extraction process yielded η = 577.6 mg (1.7% w/w) of petroleum ether extract, η = 979.6 mg (2.8% w/w) of dichloromethane extract, and η = 12665.8 mg (36.4% w/w) of methanol extract. As published previously, the extracts of *C. cinnabarinus*, *C. malicorius* (batch 2018), and *C. olivaceofuscus* were prepared by sequential soxhlet extraction [4].

For the annotation of secondary metabolites, substance‐specific parameters (i.e., molecular weight, absorption maxima, and retention time) were determined and compared with reference compounds and in‐house data [4, 5].

### 2.5 Determination of partition coefficients in two‐phase solvents systems

Partition coefficients of the seven major pigments of *C. sanguineus* in various biphasic solvent systems were determined using a modified shake‐flask method. The solvent systems were prepared in sealable 20 mL test tubes by adding selected solvents, shaking the mixture by hand, and allowing the two phases to completely separate. An aliquot of the methanolic extract (m ∼ 1 mg) was then weighed into an HPLC vial and distributed between 600 μL of each phase of the equilibrated solvent system by shaking the vial vigorously for a few seconds. After complete dissolution and equilibration, 300 μL of each phase were separately transferred into HPLC vials and dried under an air stream. The dried residues were then redissolved in DMSO (V = 500 μL) and filtered through cotton wool for subsequent HPLC analysis. The area of target peaks at λ = 400 nm was determined using the software Origin 2020. Partition coefficients were finally obtained by calculating the ratio of peak area in the lower phase (LP) divided by the peak area in the upper phase (UP). A detailed list of all tested biphasic solvent systems, including the calculated partition coefficients, is given in the appendix (Supporting Information, Chapter 4, Table S3).

### 2.6 CPC and data evaluation

Prior to each CPC run, the solvent system was freshly prepared in a separatory funnel. The solvents were thoroughly mixed and equilibrated. Subsequently, the separated organic and aqueous phases were degassed by ultrasonication for approximately 5 min. Extracts were dissolved in equal volumes of UP and LP (small scale: 750 μL each/V_total _= 1.5 mL; preparative scale: 15 mL each/V_total _= 30 mL). Before the actual separation, the CPC system was filled with two rotor volumes of stationary phase and then equilibrated by pumping the mobile phase at a chosen flow rate with a spinning rotor. The volume of the displaced stationary phase after equilibration was used to calculate the stationary phase retention volume ratio (S_f_). The biphasic solvent system chloroform/ethyl acetate/methanol/water/acetic acid = 3:1:3:2:1 v/v/v/v/v (ChEMWatAc) was applied in ascending elution mode for all CPC separations.

Analytical scale studies were performed with an approximate sample mass of 50 mg (dissolved in 750 μL UP/LP), a rotor speed of 1050 rpm, and a flow rate of 1.25 mL/min. Fractions of 2 mL (1.6 min) each were collected using a fraction collector. For evaluation, every second fraction was evaporated to dryness, reconstituted in 1 mL of DMSO, and subjected to HPLC analysis.

The large‐scale CPC separations were conducted with a sample mass of 0.7–1.0 g (dissolved in 15 mL UP/LP), a rotor speed of 1400 rpm, and a flow rate of 20 mL/min. Fractions were collected every minute (yielding 20 mL each) and analyzed by TLC utilizing two mobile phase systems (Supporting Information, Chapter 6).

Additionally, the purity of selected fractions generated in analytical scale and large‐scale experiments (Chapters 3.2, 3.3, and 3.4) was calculated and listed in the Supporting Information (Chapter 5, Table S4).

## 3 RESULTS AND DISCUSSION

AQs isolated from the basidiomycete genus *Cortinarius* have demonstrated promising photochemical and biological properties and thus indicated their potential as agents for the photodynamic treatment of infectious diseases as well as of malignant tumors [17, 18]. Fungal AQs, especially their glycosylated derivatives, however, are largely unexplored. Thus, a convenient separation technique is needed to circumvent the problematic isolation process caused, among other things, by their strong adsorption on column material [7].

**FIGURE 1:**
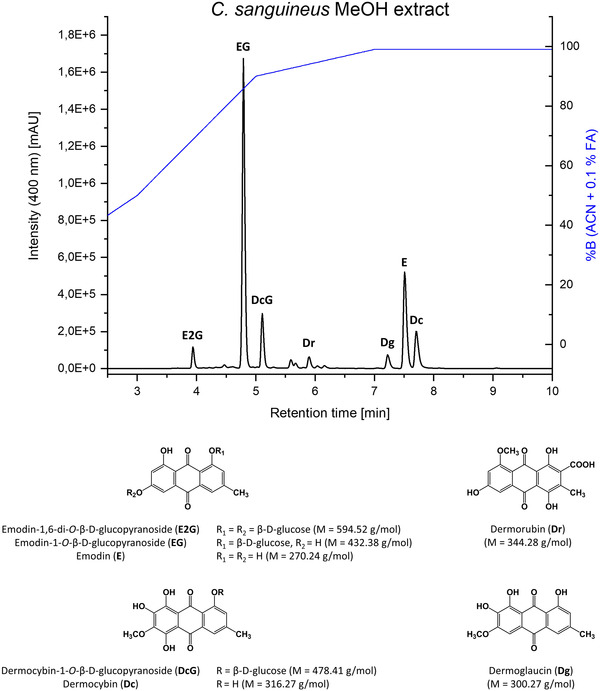
HPLC chromatogram (λ = 400 nm) of the MeOH extract (c = 10 mg/mL, DMSO) of *Cortinarius sanguineus*. The HPLC analysis was performed using a Phenomenex Synergi MAX‐RP 80 Å (150 × 4.60 mm, 4 μm) column as the stationary phase at 40°C. The mobile phase consisted of water and acetonitrile with 0.1% formic acid. The flow rate was adjusted to 1.0 mL/min and the applied gradient is indicated by the blue line. The chemical structures of annotated AQs, including their molecular weight, are shown in the lower half of the figure

Generally, fungal AQs present in the fruiting bodies of Cortinarii can be classified into regular monomeric AQs, chlorinated AQs, carboxylic acid‐substituted AQs, as well as their glycosylated derivatives. In addition, dimeric AQs and their biosynthetic precursors (i.e., pre‐AQs) also occur frequently. HPLC–DAD–MS analysis (Figure 1) of dried fruiting bodies of the Blood‐red Webcap (*C. sanguineus*) confirmed that this species produces pigments of nearly all of these AQ‐classes. Consequently, an extract of *C. sanguineus* was selected as the starting point of the method development.

### 3.1 Selection of the two‐phase solvent system for CPC separation

To ensure successful separation by CPC, selecting a suitable biphasic solvent system is imperative. The system should provide distribution coefficients (*K*_D_) for the target compounds in an acceptable range that allows them to be eluted and completely separated in a single step, i.e., *K*_D_ between 0.1 and 8 [19].

As the AQs present in the *C. sanguineus* extract cover a wide polarity range and comprise many derivatives, several classical solvent systems (Table 1) were assessed for the separation of the seven compounds depicted in Figure 1. However, neither solvent systems belonging to the hexane/ethyl acetate/methanol/water series (HEMWat, also known as the Arizona system [20]) nor to the chloroform/methanol/water (ChMWat) family yielded acceptable results. For HEMWat solvent systems, the calculation of K_D_‐values for the polar compounds **E2G**, **EG**, **DcG**, and **Dr** was not feasible because the analytes could not be detected in the UP by HPLC–DAD. The ChMWat systems showed similar behavior for the polar and even the apolar components with missing distribution between both phases. The solvent system comprising toluene, methanol, and water (4:2:4, v/v/v) successfully distributed all target metabolites. However, *K*_D_‐values were quite similar within the group of polar compounds (**E2G**, **EG**, **DcG**, and **Dr**) and within the apolar ones (**Dg** and **E**), which precluded separation by CPC. Since the regular liquid–liquid separation of fungal AQs is often achieved with the use of different acids [6], acetic acid was added to various solvent systems. The acidification of the solvent systems drastically improved the separation of all target metabolites. Of six promising acetic acid‐containing systems, the five‐component solvent system chloroform/ethyl acetate/methanol/water/acetic acid = 3:1:3:2:1 (ChEMWatAc) gave the best results, showing *K*_D_‐values in the practicable range for almost all metabolites. This system also exhibited a fast settling time and was not prone to emulsification in the presence of the fungal extract. As the *K*_D_‐values of the glycosylated compounds **E2G**, **EG**, and **DcG** were in the range of 0.1–0.9 and showed sufficient selectivity, their successful CPC separation was also anticipated to be possible in a short time. A comprehensive table of the solvent systems investigated in this study, including the calculated partition coefficients, is provided in the Supporting Information (Chapter 4, Table S3).

**TABLE 1:** Partition coefficients (*K*_D_ = Area_LP_/Area_UP_) of the seven target compounds in different biphasic solvent systems, determined using a modified shake‐flask method. *K_D_ could not be defined because no analyte was detected in the UP by HPLC, #*K*_D_ could not be defined because no analyte was detected in the lower phase by HPLC

		**Compounds and their respective *K*_D_‐values**						
**Solvent system**	**Composition (v/v)**	**E2G**	**EG**	**DcG**	**Dr**	**Dg**	**E**	**Dc**
Toluene/methanol/water	4:2:4	2.11	2.00	1.87	2.03	0.41	0.44	0.62
Hexane/ethyl acetate/methanol/water	6:4:5:5	*	*	*	*	0.21	0.24	8.72
Hexane/ethyl acetate/methanol/water	9:1:5:5	*	*	*	*	6.82	0.63	22.0
Chloroform/methanol/water	10:4:6	#	5.1E‐2	#	#	*	*	*
Chloroform/methanol/water	10:3:7	#	3.0E‐2	#	#	*	*	*
Chloroform/methanol/water/acetic acid	4:3:2:1	8.5E‐2	0.75	1.25	4.44	16.0	12.7	16.4
Chloroform/methanol/water/acetic acid	4:3.5:2:0.5	0.13	0.82	1.22	2.38	32.1	18.1	19.8
Chloroform/methanol/water/acetic acid	4:3:2.5:0.5	2.0E‐2	0.63	1.14	2.44	14.5	19.8	31.6
Chloroform/ethyl acetate/methanol/water/acetic acid	3:1:3.5:2:0.5	9.2E‐2	0.60	0.77	2.20	16.2	13.7	13.6
Chloroform/ethyl acetate/methanol/water/acetic acid	3:1:3:2:1	0.18	0.70	0.84	1.79	7.32	7.77	8.51

### 3.2 CPC separation of the Cortinarius sanguineus methanolic extract

**FIGURE 2:**
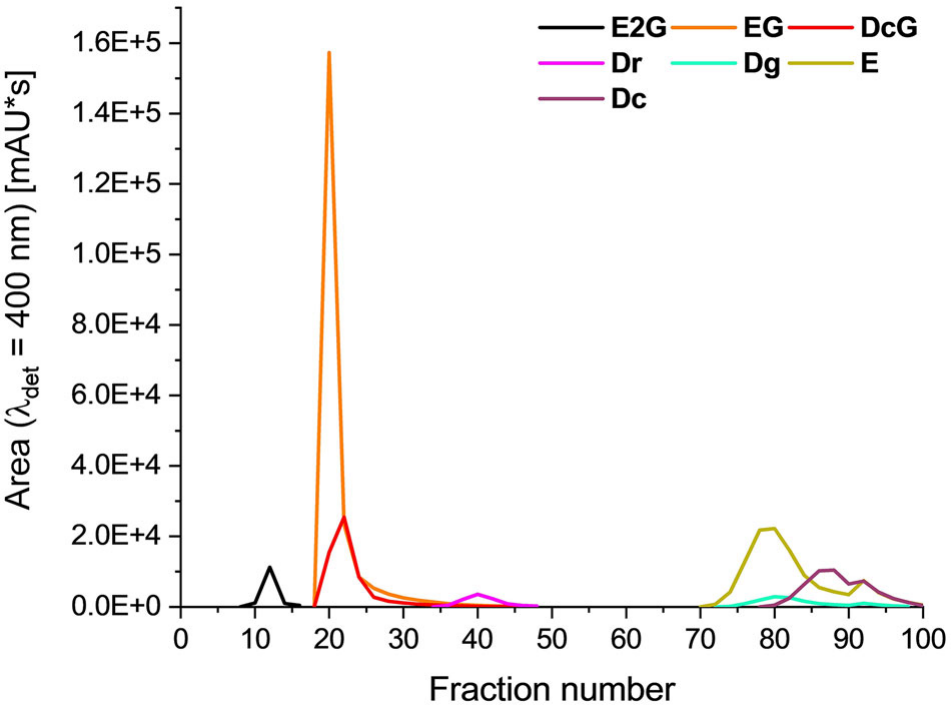
Fractogram of the seven target compounds from the CPC fractionation of the C. sanguineus methanolic extract (sample concentration: 50 mg in 1.5 mL upper/lower phase mixture). The experiment was conducted on the 55 mL rotor, with rotor speed and flow rate adjusted to 1050 rpm and 1.25 mL/min, respectively

CPC separation on an analytical scale was carried out using the ChEMWatAc solvent system in ascending mode, i.e., the lower phase served as the stationary phase and the UP as the mobile phase. A rotation speed of 1050 rpm combined with a flow rate of 1.25 mL/min allowed for low column bleeding, a stable stationary phase retention volume ratio (S_f_) of 56% after equilibration, and a maximum backpressure of 27 bar. Studies to optimize the parameters have shown that a reduced rotation speed led to an increased loss of stationary phase, while a further increase in speed led to a build‐up of heat in the device (data not shown). An aliquot of the *C. sanguineus* methanolic extract (m = 61.3 mg) was dissolved in 750 μL of each phase (V_total_ = 1.5 mL) and injected into the 5 mL sample loop. Fractions of 2 mL were collected, and every second fraction was subjected to HPLC analysis. After manual integration of compound peaks, a fractogram was created to illustrate the separation capability of the tested system (Figure 2).

Complete fractionation of the extract was achieved within 100 min. As depicted Figure 2, the aim—facilitating the purification of AQ‐glucopyranosides—was successfully achieved: **E2G**, the diglucopyranoside of emodin, was completely separated from the other pigments. **EG** and **DcG** partially co‐eluted; however, a clear enrichment of each compound can be realized by a strategic combination of the fractions. **Dr**, belonging to the class of AQ carboxylic acids, was entirely separated from the glycosylated and monomeric AQs. Though not fully resolved, the three monomeric AQs **Dg**, **E**, and **Dc** were successfully separated from all other pigments. In detail, **Dg** and a large part of **E** eluted first, followed by the majority of **Dc**.

In sum, CPC separation with the ChEMWatAc system fractionated the complex AQ‐rich extract according to chemical classes (i.e., diglycosylated AQs, monoglycosylated AQs, AQs with acidic functions, and regular monomeric AQs) and showed selectivity within these classes.

### 3.3 Applicability of the separation technique to *Cortinarius* species with different pigmentation types

**FIGURE 3:**
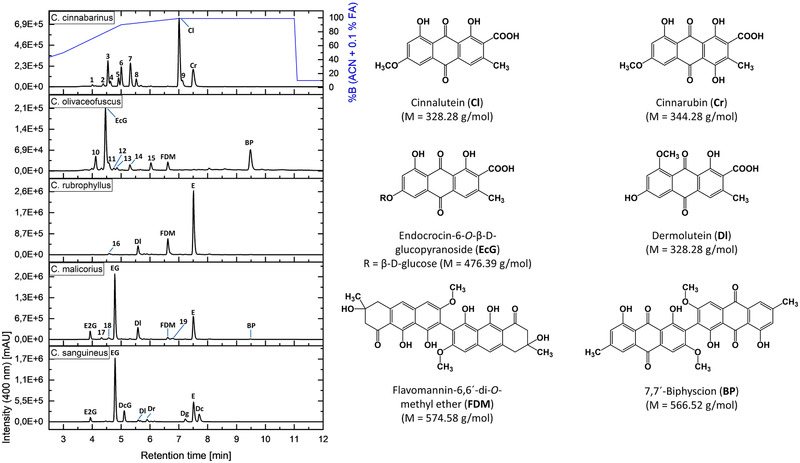
HPLC chromatograms (λ = 400 nm) of Cortinarius species with different pigmentation types (MeOH extracts, c = 10 mg/mL, DMSO). Chemical structures of annotated AQs, including their molecular weight, are depicted on the right side (compounds **1**–**19** are without reliable identification). The HPLC analysis was performed using a Phenomenex Synergi MAX‐RP 80 Å (150 × 4.60 mm, 4 μm) column as the stationary phase at 40°C. The mobile phase consisted of water and acetonitrile with 0.1% formic acid. The flow rate was adjusted to 1.0 ml/min and the applied gradient is indicated by the blue line

Already in 1968, a TLC‐based analysis of various colorful fungi belonging to the subgenus *Dermocybe* led to the classification of four pigmentation types (PTs) based on their pigment patterns (i.e., cinnabarina, cinnamomea, malicoria, and sanguinea PT) [1]. Motivated by the promising fractionating capabilities of the CPC system for the sanguinea PT (*C. sanguineus*, Chapter 3.2), its value for other PTs was evaluated. Thus, extracts of *C. cinnabarinus* (cinnabarina PT), *C. olivaceofuscus* (cinnamomea PT), *C. rubrophyllus* (malicoria PT), and *C. malicorius* (malicoria PT) were submitted to an analytical scale CPC separation. Prior to this, however, their pigments were analyzed by HPLC–DAD–MS and annotated by comparison with authentic reference samples (Figure 3). Peaks that did not yield a reliable annotation due to insufficient reference data or low concentrations were labeled with numbers (**1–19**).

**FIGURE 4:**
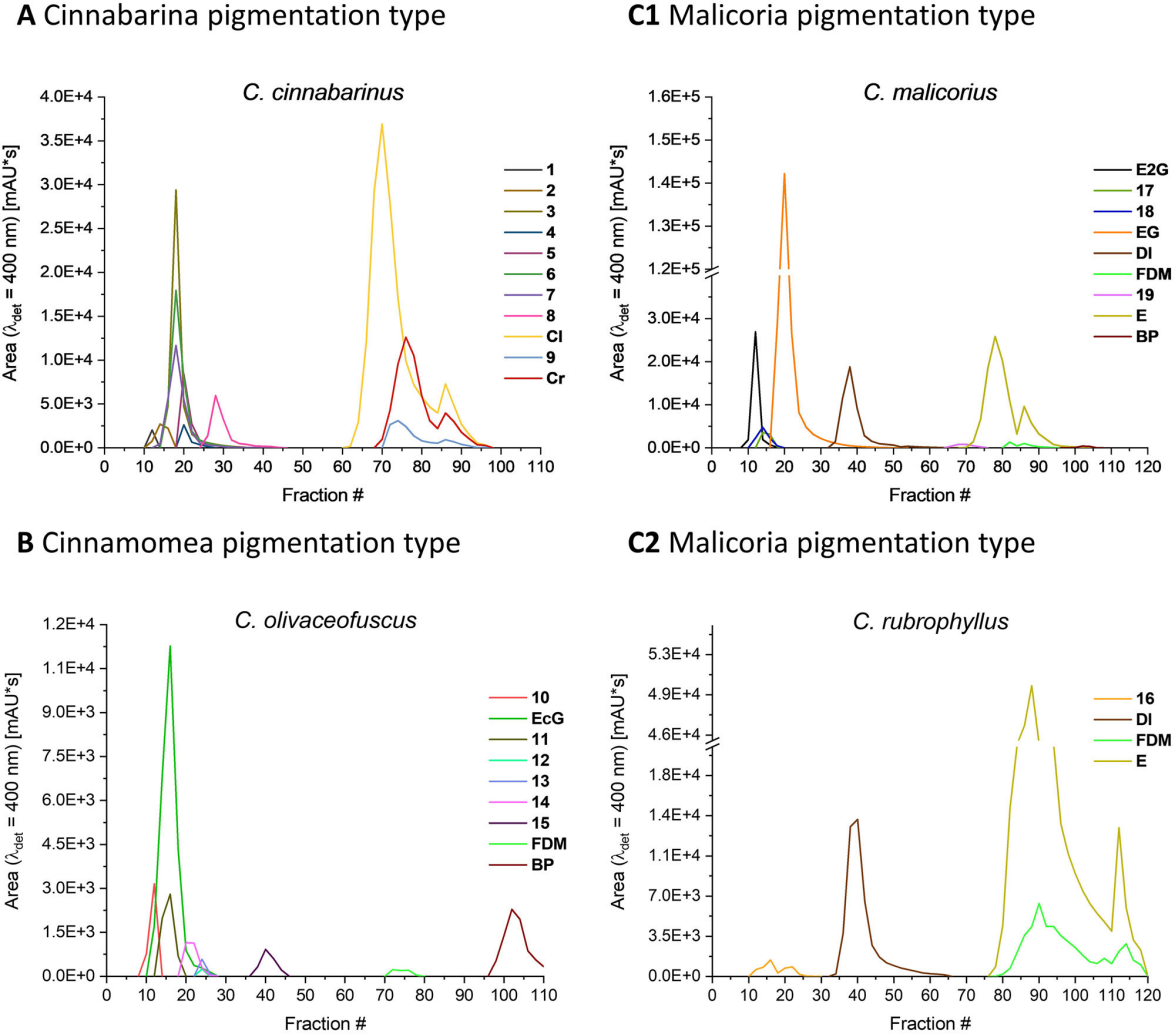
Fractograms obtained through the CPC separation of four fungal extracts with different pigmentation types. For all four experiments, a sample concentration of about 50 mg in 1.5 mL upper/lower phase mixtures was chosen. The experiments were performed on the 55 mL rotor with a rotor speed of 1050 rpm and a flow rate of 1.25 mL/min

Aliquots (m ∼ 50 mg) of methanolic extracts of the four Cortinarii species were separated on an analytical scale employing the ChEMWatAc system. Again, a fractogram was generated by examining every second fraction. The CPC separation of the *C. cinnabarinus* extract (cinnabarina PT) showed that the polar compound **8** and the majority of cinnalutein (**Cl**) could be fully resolved from the highly complex mixture of pigments (Figure 4A). Unfortunately, the other metabolites were not separated. *C. cinnabarinus* produces many AQ carboxylic acids, such as cinnalutein (**Cl**) and cinnarubin (**Cr**), with similar chemical backbones, including their sugar‐substituted derivatives [13]. Thus, the relatively weak separating power of the presented ChEMWatAc system is not surprising, as it was designed to separate the general pigment classes.

Belonging to the cinnamomea PT, *C. olivaceofuscus* lacks the orange pigment emodin and its glycosylated derivatives [1]. Fungi exhibiting this specific PT produce high amounts of pre‐AQs (e.g., **FDM**), which are biosynthetic precursors of dimeric AQs like 7,7´‐biphyscion (**BP**) [13]. Separation with the ChEMWatAc system (Figure 4B) showed that the polar compounds (i.e., **10–14** and **EcG**) were partially co‐eluted and three pigments **15**, **FDM**, and **BP** were fully separated. Thus, rapid isolation of the promising photosensitizer **BP** [4] is achievable through CPC separation from a crude extract.

Fungi of the malicoria PT are usually orange in color and contain emodin and its glucopyranoside as main pigments [1]. As in cinnamomea PT‐fungi, pre‐AQs can also occur (Figure 3). The presented system for fractionation was used on a *C. malicorius* extract obtained with Soxhlet extraction, thus on an extract with only a few pre‐AQs (Figure 4C1). First, the diglucopyranoside of emodin (**E2G**) eluted and was fully resolved from the monoglucopyranoside (**EG**). Pigments **17** and **18** co‐eluted between the emodin derivatives. The AQ carboxylic acid dermolutein (**Dl**) was obtained just after **EG**. Peak **19** was eluted in fractions 66–74. Following peak **19**, the elution of the majority of **E** was observed. **FDM** was found to partially co‐elute with **E**. Lastly, small amounts of **BP** were detected in the fractions 98–106. Hence, not only all four main components of the extract (i.e., **E2G**, **EG**, **Dl**, and **E**) were quantitatively separated from each other, but also two minor compounds (Peak **19** and **BP**) were obtained in the absence of other pigments. CPC separation of an extract rich in pre‐AQs was tested with a methanolic extract of *C. rubrophyllus* (Figure 4C2). The results for both malicoria PT‐fungi were largely comparable.

Summarizing the results of the analytical investigations on the applicability of the ChEMWatAc system to complex extracts with various pigmentation patterns, it can be said that multiple fungal AQs can be easily separated, as good selectivity was observed towards and within the different subgroups of AQs. The CPC separation of the *C. cinnabarinus* extract gave the least impressive result with two pigments separated. In contrast, all the major pigments were separated in a single step in the *C. malicorius* extract.

### 3.4 Large‐scale CPC separation of the methanolic extracts of *C. malicorius* (malicoria PT) and *C. sanguineus* (sanguinea PT)

**FIGURE 5:**
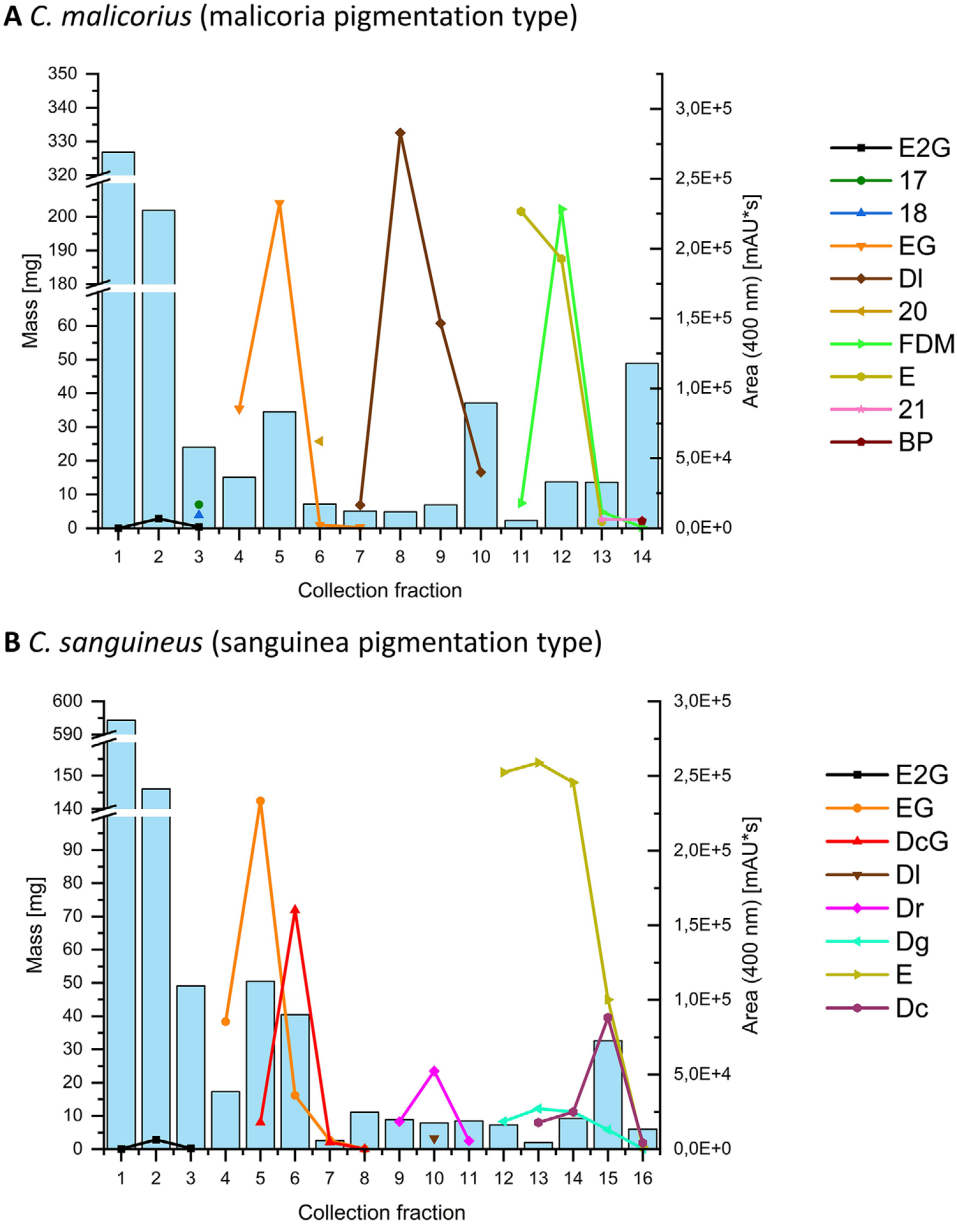
Fractograms obtained through the CPC separation of the methanolic extracts of C. malicorius (A) and C. sanguineus (B). On the *x*‐axis the number of the collection fraction, on the left y‐axis the mass of the respective fraction. On the right *y*‐axis, the peak area of peaks detected in the respective fractions is displayed (compounds 17, 18, 20, and 21… pigments without reliable identification). Both separations were conducted on the 1000 ml rotor, with rotor speed and flow rate set to 1400 rpm and 20 ml/min, respectively

A large‐scale CPC employing the ChEMWatAc system was carried out in ascending mode, with a rotation speed of 1400 rpm, and a flow rate of 20 mL/min. The parameters gave a stable stationary phase retention volume ratio (S_f_) of 53% and a backpressure of 47 bar. The methanolic extract of *C. sanguineus*, which was used to develop the system, and an extract prepared via ultrasonication from a relatively fresh batch of *C. malicorius* fruiting bodies were used in this investigation. Aliquots of the extracts (m_Csan_ = 1014.7 mg, m_Cmal_ = 745.2 mg) were dissolved in equal volumes of mobile and stationary phase (V_total _= 30 mL) and injected into the 50 mL sample loop. Fractions of 20 mL each were collected and every second one was analyzed by TLC. Representatives of the TLC analysis are provided in the supplementary material (Chapter 6, Figures S1 and S2). Thereafter, respective fractions were combined, and a fractogram for each extract was generated, displaying the masses of the collected fractions and the peak areas of pigments occurring within (Figure 5). Stationary phase extrusion was performed and monitored via TLC so that the majority of the used extracts were recovered (recovery rates for both extracts > 95%).

The first fractions obtained from both extracts had a large mass but did not contain compounds capable of absorbing visible light (Figure 5A and B). Since the extracts were prepared with a polar solvent and Cortinarii are known to produce high amounts of various sugars [21], it was assumed that these fractions primarily contained glycosides. A proton NMR experiment confirmed this hypothesis (Figure S4 and S5). Thus, a large part of unwanted metabolites was separated from the target pigments already. Just like in the previous experiment, larger‐scale CPC of the *C. malicorius* extract achieved separation of all major compounds, i.e., **E2G**, **EG**, **20**, **Dl**, and **E**. Fractions were obtained that contained mainly **E** (Figure 5A, collection fraction 11) as well as **E** and **FDM** in almost equal amounts (collection fraction 12). **BP** was detected in collection fraction 14.

When assaying the methanolic extract of *C. sanguineus*, the polar metabolites eluted in the expected order, i.e., **E2G**, **EG**, and **DcG**. Compared to the analytical‐scale experiment, an enhanced separation of **EG** and **DcG** was observed. Complete separation and subsequent purification of these compounds would be possible with another liquid–liquid separation using ethyl acetate/water as an extracting agent for **EG** followed by simple recrystallization from aqueous ethanol [6]. As shown in Figure 5B (collection fractions 9–11), even the fractionation of the group of AQ carboxylic acids was achieved. Two fractions were obtained that contained only **Dr** and one fraction that contained **Dr** and **Dl**. Fraction 12 consisted of **E** with some minor amounts of **Dg**. Complete separation of these compounds is possible through column chromatography on silica gel, where **E** can be eluted from the column with dichloromethane. **Dg** is retained under these conditions and can be eluted selectively with acidified acetone [6]. Since the majority of **E** and also of **Dg** had already eluted in fractions 12–14, fraction 15 consisted of large quantities of **Dc**. A protocol for the purification of **Dc** tainted by **E** and **Dg** can be found elsewhere (SI, Chapter 9) [6].

## 4 CONCLUDING REMARKS

The recent discovery of photoactivity in basidiomycetes has reignited the interest in naturally occurring AQs. A novel CPC technique was developed to facilitate the convenient and rapid separation of these promising photosensitizers. The **ChEMWatAc** solvent system was demonstrated to efficiently separate crude extracts of various *Cortinarius* species containing AQs (e.g., species of *Cortinarius* subgenus *Dermocybe*). When applied to fungi with different PTs, CPC separated two pigments (*C. cinnabarinus*) in the worst case and six pigments (*C. malicorius*) in the best case. Considering the complex composition of the extracts used and the excellent separation performance of the presented technique, this strategy makes an essential contribution to a rapid separation process for extracts from a wide range of *Cortinarius* species and very likely of other fungal taxa containing AQs. The large‐scale experiments proved that scale‐up is possible without further complications (e.g., decreased selectivity). This study clearly emphasizes the versatility of liquid–liquid‐based separation techniques such as CPC to separate fungal AQs.

## CONFLICT OF INTEREST

The authors have declared no conflict of interest.

## Supporting information

Supporting Information

## Data Availability

The data that supports the findings of this study are available in the supplementary material of this article.
