# Supplementary material for: A convenient separation strategy for fungal anthraquinones by centrifugal partition chromatography
Source: J Sep Sci. 2022 Jan 19;45(5):1031–41. doi: 10.1002/jssc.202100869 (PMC9415121; doi:10.1002/jssc.202100869)
Supplement: Supplementary file 1 — Supporting Information [file JSSC-45-1031-s001.docx]

Electronic supplementary information (SI)

A convenient separation strategy for fungal anthraquinones by centrifugal partition chromatography

Fabian Hammerle^1^, Michael Zwerger^1^, Anja Höck^1^, Markus Ganzera^1^, Ursula Peintner^2^, Bianka Siewert^1*^

1 Institute of Pharmacy/Pharmacognosy, Center for Molecular Biosciences Innsbruck, University of Innsbruck, Innsbruck, Austria

2 Institute of Microbiology, University of Innsbruck, Innsbruck, Austria

Correspondence: Dr. Bianka Siewert, Institute of Pharmacy/Pharmacognosy, Center for Molecular Biosciences Innsbruck, University of Innsbruck, Innrain 80/82, 6020 Innsbruck, Austria, Email: [bianka.siewert@uibk.ac.at](mailto:bianka.siewert@uibk.ac.at)

Keywords (alph. order, max. 5): anthraquinone glycosides, anthraquinones, basidiomycetes, centrifugal partition chromatography, Cortinarius

Funding information: Austrian Science Fund (FWF, P31915)

**Abstract:**

As recently shown, some fungal pigments exhibit significant photoactivity turning them into promising agents for the photodynamic treatment of microbial infections or malignant diseases. In the present study, a separation strategy for fungal anthraquinones was developed based on centrifugal partition chromatography. A suitable method was explored employing a methanolic extract of the fruiting bodies of *Cortinarius sanguineus* (Agaricales, Basidiomycota). An excellent fractionation was achieved using a biphasic solvent system comprising chloroform/ethyl acetate/methanol/water/acetic acid (3:1:3:2:1 v/v/v/v/v) operating in ascending mode. Experiments on an analytical scale with extracts of closely related *Cortinarius* species exhibited broad applicability of the devised system. Up to six pigments could be purified directly from the crude extract. Preparative-scale fractionation of the methanol extracts of *C. malicorius* and *C. sanguineus* demonstrated that up-scaling was possible without compromising selectivity.

Table of contents

[2 Fungal material 3](#_Toc89430343)

[3 Extract yields 3](#_Toc89430344)

[4 Partition coefficients 5](#_Toc89430345)

[5 Purity estimation of selected fractions 7](#_Toc89430346)

[6 TLC-analysis – CPC large-scale experiments 10](#_Toc89430347)

[*6.1* *Cortinarius malicorius* 10](#_Toc89430348)

[*6.2* *Cortinarius sanguineus* 11](#_Toc89430349)

[7 HPLC-DAD analysis *C. malicorius* extract 12](#_Toc89430350)

# Fungal material

Voucher material of the investigated *Cortinarius* species is deposited in the Mycological Collection of the Tiroler Landesmuseen (IBF).

**Table S1.** Cortinarius species used in this study with their respective voucher numbers and collection data.

| **Species** | **Voucher** | **leg. et det.** | **Origin** | **Date of Collection** |
| --- | --- | --- | --- | --- |
| *C. cinnabarinus* Fr. | IBF19801005 | R. Pöder | Sweden, Småland, Femsjö | 17.09.1980 |
| *C. malicorius* Fr. | IBF20180009 | D. Borghi | Italy, South Tyrol, Sexten | 15.08.2018 |
| *C. malicorius* Fr. | IBF20200054 | L. Huymann | Austria, Tyrol, Lans | 21.09.2020 |
| *C. olivaceofuscus* Kühner (*D. carpineti* nom. inval.) | IBF19750023 | M. Moser | Switzerland, Basel, Dreiländereck | 12.07.1975 |
| *C. rubrophyllus* (Moënne-Locc.) Liimat., Niskanen, Ammirati & Dima | IBF20190002b GenBank Acc. MZ357345 | L. Huymann | Austria, Tyrol, Mutters | 22.08.2019 |
| *C. sanguineus var. aurantiovaginatus* Fillion & Moënne-Locc. | IBF20200038  GenBank Acc. MW880281 | L. Huymann | Austria, Tyrol, Mutters | 02.10.2020 |

# Extract yields

**Table S2.** Overview of the extract yields obtained with different extraction methods (i.e., Soxhlet extraction and ultra-sonication).

| **Soxhlet extraction** | **Mass biomaterial [g]** | **Solvent** | **Yield [mg (%dw)]** |
| --- | --- | --- | --- |
| C. cinnabarinus | 2.08 | Petroleum ether | 37 (1.8 %) |
|  |  | Methaol | 775 (37.2 %) |
| C. malicorius | 2.08 | Petroleum ether | 22 (1.1 %) |
|  |  | Methanol | 666 (31.9 %) |
| C. olivaceofuscus | 2.06 | Petroleum ether | 15 (0.7 %) |
|  |  | Methanol | 687 (33.3 %) |
| **Ultra-sonication** |  |  |  |
| C. malicorius | 6.01 | Methanol | 1267 (21.1 %) |
| C. rubrophyllus | 0.93 | Methanol | 352 (37.8 %) |
| C. sanguineus | 34.80 | Petroleum ether | 578 (1.7 %) |
|  |  | Dichloromethane | 980 (2.8 %) |
|  |  | Methanol | 12666 (36.4 %) |

# Partition coefficients

**Table S3.** Partition coefficients (K_D_ = Area_LP_/Area_UP_) of E2G, EG, DcG, Dr, Dg, E, and Dc in various solvent systems, determined using the shake-flask method. * … K_D_ could not be defined because no analyte was detected in the upper phase by HPLC, # … K_D_ could not be defined because no analyte was detected in the lower phase by HPLC.

|  |  | **Compounds and their respective K_D_ values** | | | | | | | |
| --- | --- | --- | --- | --- | --- | --- | --- | --- | --- |
| **Solvent system** | **Composition (v/v)** | **E2G** | **EG** | **DcG** | **Dr** | **Dg** | **E** | **Dc** |  |
| hexane/ethanol/water | 4:3.5:2.5 | * | * | * | * | * | 6.73 | * |  |
| hexane/ethanol/water | 1.8:2.2:0.3 | * | * | * | * | * | 15.9 | * |  |
| heptane/methanol/water | 4:3:3 | * | 266 | * | * | * | 4.16 | * |  |
| hexane/ethyl acetate/methanol/water | 3:7:5:5 | * | 16.4 | * | * | 0.22 | 0.13 | 2.65 |  |
| hexane/ethyl acetate/methanol/water | 5:5:5:5 | * | 62.7 | 114 | * | 1.06 | 0.50 | 8.95 |  |
| ethyl acetate/ethanol/water | 4:1:5 | 6.38 | 0.65 | 8.36 | 16.6 | # | 1.7E-2 | 0.23 |  |
| ethyl acetate/methanol/water | 4.5:1:4.5 | 7.54 | 0.91 | 8.48 | * | 0.15 | 0.21 | 0.38 |  |
| chloroform/acetic acid/water | 3.5:2.5:4 | # | 2.5E-2 | 6.3E-2 | 0.40 | * | 30.2 | 16.2 |  |
| chloroform/methanol/water | 4:3:2 | # | 0.23 | # | # | * | 13.7 | 1.53 |  |
| chloroform/methanol/water | 10:5:5 | # | 0.10 | # | # | * | 26.3 | 5.29 |  |
| chloroform/ethanol/water | 4:2:4 | # | 0.41 | # | # | * | * | * |  |
| *n*-butanol/methanol/water | 4.5:0.5:5 | 0.85 | 0.11 | 1.03 | 2.47 | # | 9.5E-3 | 3.9E-2 |  |
| chloroform/ethyl acetate/*n*-butanol/water/acetic acid | 3:2:1:3:1 | 1.9E-3 | 0.40 | 0.39 | 18.3 | 66.3 | 48.7 | 60.2 |  |
| chloroform/ethyl acetate/*n*-butanol/water/acetic acid | 2:2:1:4:1 | # | 1.25 | 1.14 | * | * | 96.9 | 126 |  |

# Purity estimation of selected fractions

**Table S4.** Estimation of the purity of selected CPC fractions obtained through analytical as well as large-scale experiments. The fractions were analysed using HPLC-DAD and integrated with Origin 2020. Purity was calculated as the quotient of "peak area of target compound" and "sum of peak areas of all detected peaks" (n.d. … not detected).

|  |  |  | **Purity @ respective wavelength [%]**  **= (Peak area of target compound)/(Sum of peak areas of all detected peaks) x 100** | | | | |
| --- | --- | --- | --- | --- | --- | --- | --- |
| **Fungal extract** | **CPC fraction** | **Target compound(s)** | **254 nm** | **366 nm** | **400 nm** | **430 nm** | **470 nm** |
| **Analytical scale** | | | | | | | |
| *C. sanguineus* | 12 | **E2G** | 41.3 | 73.8 | 88.5 | 90.8 | 76.4 |
|  | 18 | **EG** | 83.8 | 98.6 | 99.5 | 99.3 | 97.5 |
|  | 20 | **EG \| DcG** | 65.8 \| 32.8 | 85.6 \| 14.3 | 91.0 \| 9.0 | 87.3 \| 12.7 | 51.3 \| 48.7 |
|  | 22 | **EG \| DcG** | 35.5 \| 63.8 | 39.9 \| 59.9 | 47.8 \| 51.9 | 39.5 \| 60.2 | 10.7 \| 89.3 |
|  | 40 | **Dr** | 50.0 | 46.6 | 62.4 | 62.2 | 84.5 |
|  | 78 | **Dg \| E** | 4.7 \| 94.8 | 6.2 \| 93.7 | 8.1 \| 91.9 | 6.6 \| 93.4 | 4.6 \| 95.0 |
|  | 88 | **E \| Dc** | 18.0 \| 81.7 | 24.6 \| 73.0 | 27.8 \| 68.3 | 27.1 \| 69.9 | 11.9 \| 87.5 |
| *C. cinnabarinus* | 28 | **8** | 70.4 | 73.9 | 77.8 | 81.2 | 80.5 |
|  | 68 | **Cl** | 98.5 | 98.5 | 99.8 | 99.8 | 97.3 |
| *C. olivaceofuscus* | 40 | **15** | 50.8 | 76.3 | 100.0 | 100.0 | 98.1 |
|  | 76 | **FDM** | 100.0 | 100.0 | 100.0 | 100.0 | n.d. |
|  | 104 | **BP** | 88.2 | 95.0 | 96.0 | 95.6 | 95.4 |
| *C. malicorius* | 12 | **E2G** | 51.3 | 61.9 | 91.4 | 98.8 | 97.3 |

**Table S4 [Continued].** Estimation of the purity of selected CPC fractions obtained through analytical as well as large-scale experiments. The fractions were analysed using HPLC-DAD and integrated with Origin 2020. Purity was calculated as the quotient of "peak area of target compound" and "sum of peak areas of all detected peaks" (n.d. … not detected).

|  |  |  | **Purity @ respective wavelength [%]**  **= (Peak area of target compound)/(Sum of peak areas of all detected peaks) x 100** | | | | |
| --- | --- | --- | --- | --- | --- | --- | --- |
| **Fungal extract** | **CPC fraction** | **Target compound(s)** | **254 nm** | **366 nm** | **400 nm** | **430 nm** | **470 nm** |
| *C. malicorius* | 20 | **EG** | 87.8 | 95.8 | 99.6 | 99.8 | 99.5 |
|  | 38 | **Dl** | 86.2 | 89.9 | 94.7 | 95.2 | 93.9 |
|  | 68 | **19** | 88.0 | 95.8 | 94.9 | 91.6 | 95.8 |
|  | 78 | **E** | 98.0 | 96.9 | 96.7 | 99.3 | 99.8 |
| *C. rubrophyllus* | 16 | **16** | 49.0 | 64.7 | 75.5 | 76.6 | n.d. |
|  | 38 | **Dl** | 65.9 | 68.1 | 95.5 | 95.8 | 90.1 |
|  | 82 | **E** | 96.4 | 94.9 | 94.4 | 98.9 | 99.8 |
| **Large-scale** | | | | | | | |
| *C. malicorius* | 5  (m = 34.5 mg) | **EG** | 85.5 | 99.6 | 99.3 | 99.2 | 99.5 |
|  | 6  (m = 7.1 mg) | **20** | 88.0 | 85.1 | 96.5 | 98.5 | 93.8 |
|  | 8  (m = 4.8 mg) | **Dl** | 79.4 | 95.5 | 97.2 | 96.8 | 96.4 |
| *C. malicorius* | 11  (m = 13.7 mg) | **FDM \| E** | 9.3 \| 70.9 | 6.0 \| 83.0 | 5.3 \| 89.3 | 2.1 \| 96.7 | n.d. \| 99.3 |

**Table S4 [Continued].** Estimation of the purity of selected CPC fractions obtained through analytical as well as large-scale experiments. The fractions were analysed using HPLC-DAD and integrated with Origin 2020. Purity was calculated as the quotient of "peak area of target compound" and "sum of peak areas of all detected peaks" (n.d. … not detected).

| *C. sanguineus* | 4  (m = 17.3 mg) | **EG** | 60.7 | 99.2 | 99.3 | 99.4 | 98.8 |
| --- | --- | --- | --- | --- | --- | --- | --- |
|  | 5  (m = 50.5 mg) | **EG \| DcG** | 69.3 \| 30.2 | 92.6 \| 7.4 | 92.9 \| 7.4 | 89.5 \| 10.5 | 65.5 \| 34.4 |
|  | 6  (m = 40.4 mg) | **EG \| DcG** | 27.6 \| 64.9 | 17.5 \| 82.0 | 18.3 \| 81.4 | 16.3 \| 83.4 | 4.6 \| 95.4 |
|  | 12  (m = 7.3 mg) | **Dg \| E** | 10.3\| 79.8 | 5.0 \| 94.3 | 6.8 \| 92.8 | 7.5 \| 90.7 | 3.5 \| 93.4 |
|  | 15  (m = 32.6 mg) | **Dg \| E \| Dc** | 4.4 \| 39.1 \| 56.5 | 4.4 \| 45.3 \| 50.3 | 6.1 \| 49.4 \| 43.9 | 3.9 \| 48.6 \| 47.5 | 1.3 \| 27.1 \| 71.6 |

# TLC-analysis – CPC large-scale experiments

## *Cortinarius malicorius*


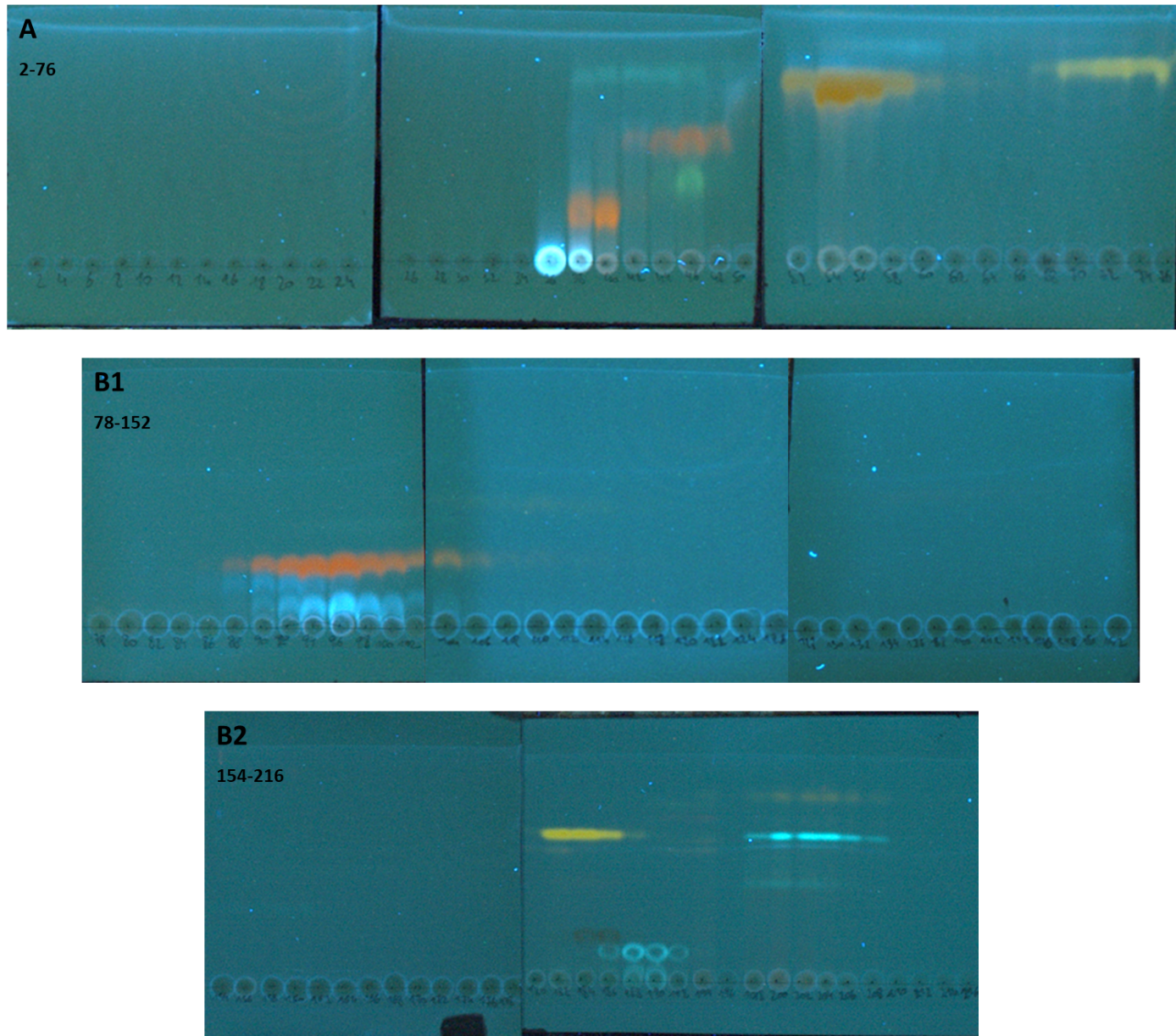


**Figure S1.** TLC analysis of fractions obtained through the large-scale CPC separation of the C. malicorius MeOH extract (UV 366 nm). Mobile phase: A – toluene/acetone/formic acid/acetic acid (35:40:12.5:12.5 v/v/v/v), B1&B2 – toluene/ethyl acetate/formic acid/acetic acid (70:20:5:5 v/v/v/v).

## *Cortinarius sanguineus*


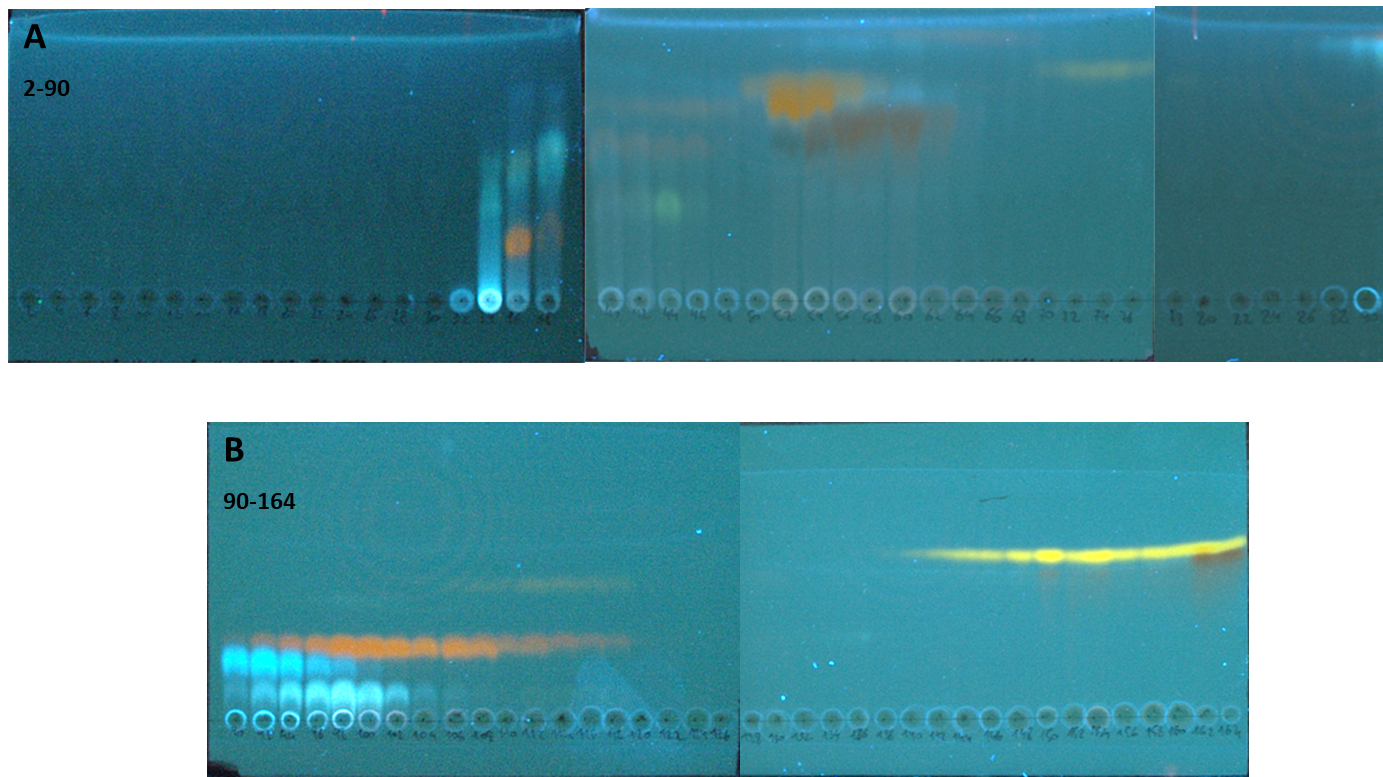


**Figure S2.** TLC analysis of fractions obtained through the large-scale CPC separation of the C. sanguineus MeOH extract (UV 366 nm). Mobile phase: A – toluene/acetone/formic acid/acetic acid (35:40:12.5:12.5 v/v/v/v), B – toluene/ethyl acetate/formic acid/acetic acid (70:20:5:5 v/v/v/v).

# HPLC-DAD analysis *C. malicorius* extract


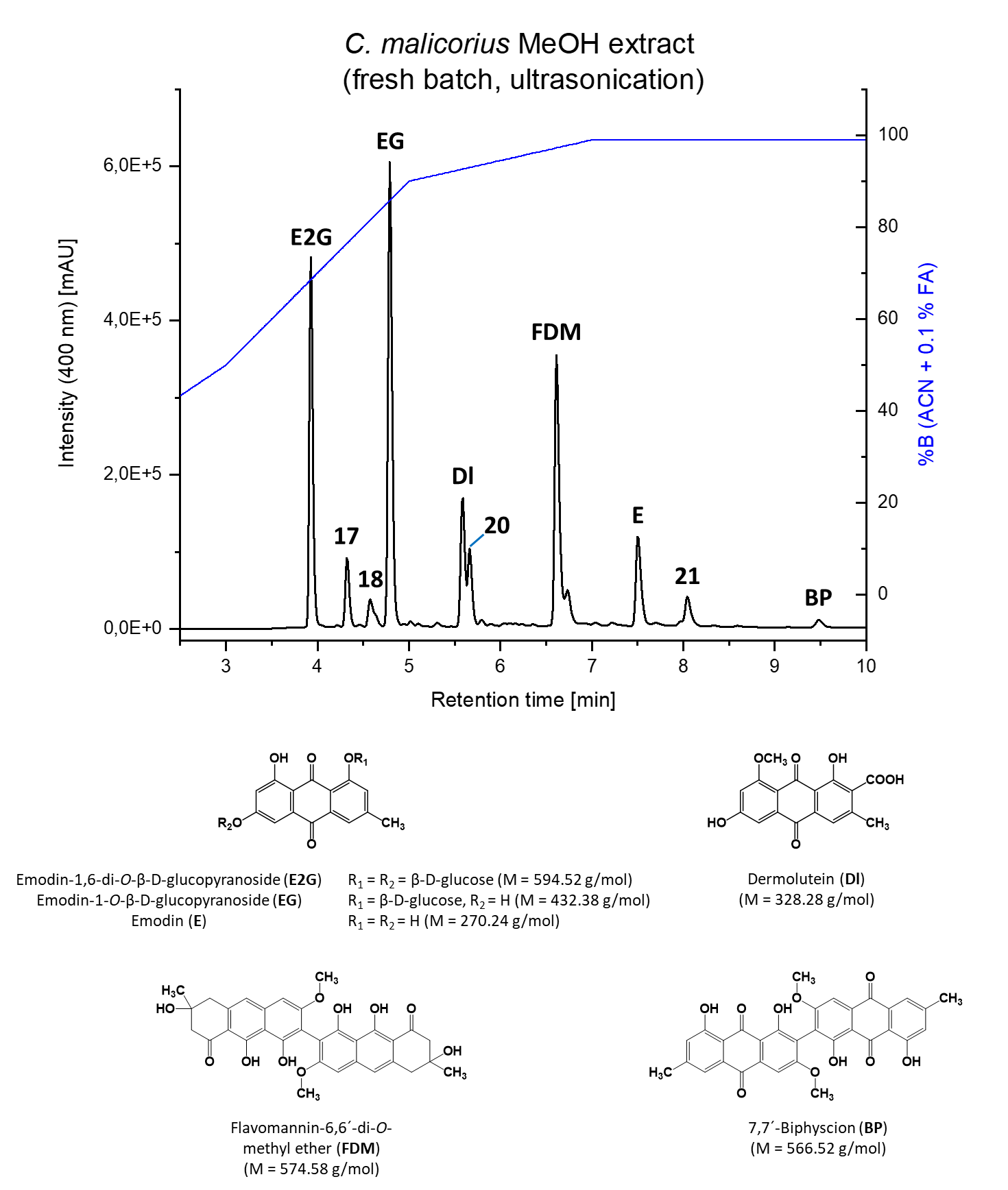


**Figure S3.** Chromatogram (λ = 400 nm) obtained through the HPLC-DAD-MS analysis of the C. malicorius MeOH extract. Annotated compounds are provided with indicators above the corresponding peaks. Chemical structures of the annotated compounds are depicted below the chromatogram. For peaks with numeric indicators, a reliable identification was not possible (i.e., peaks **17**, **18**, **20**, and **21**).
